# Supplementary material for: Patients’ perceptions of home-based imaging diagnostics: A qualitative study
Source: PLoS One. 2026 Feb 27;21(2):e0343607. doi: 10.1371/journal.pone.0343607 (PMC12948074; doi:10.1371/journal.pone.0343607)
Supplement: S1 File — Script and questions. (DOCX) [file pone.0343607.s001.docx]

**SCRIPT AND QUESTIONS**

**Patients’ Perceptions of Home Diagnostic Imaging Services:**

- How old are you?
- What gender do you identify with?
- What imaging diagnostic exam(s) did you perform with the company?
- Do you prefer to perform imaging diagnostic exams at home or at the clinic? Why?
- How did you prepare for the diagnostic exam? Was it easy, normal or difficult?
- In your opinion, what are the positive aspects of being able to perform this diagnostic exam at home? (points that stood out positively, that you liked)
- In your opinion, what are the negative points of performing this diagnostic exam at home? (weak points that you didn't like or that should be improved)
- Are you concerned about the service being performed at home?
- Do you trust the exam that was performed? Why?
- What do you think of the results? Better than the clinic, the same, worse, or do you not know how to answer?
- Overall, do you feel that you received good care from the professionals during the home visit to take the exam? Why?
- Were the professionals well dressed and equipped?
- Did the professionals arrive at the correct time (were they punctual)?
- Did you feel that healthcare professionals were attentive and answered your questions?
- What is your opinion about the equipment used to perform the exam?
- Are they modern?
- Were they in good condition?
- What is your opinion about the transport vehicle used by the company to bring you home?
- Was it in good condition?
- Would you change anything about the way the service was performed?
